# Incorporation of DNA methylation quantitative trait loci (mQTLs) in epigenome-wide association analysis: application to birthweight effects in neonatal whole blood

**DOI:** 10.1186/s13148-022-01385-6

**Published:** 2022-12-01

**Authors:** Shaobo Li, Nicholas Mancuso, Catherine Metayer, Xiaomei Ma, Adam J. de Smith, Joseph L. Wiemels

**Affiliations:** 1 Department of Population and Public Health Sciences, Center for Genetic Epidemiology, University of Southern California, USC Health Sciences Campus, 1520 San Pablo St., Los Angeles, CA USA; 2 School of Public Health, University of California Berkeley, Berkeley, CA USA; 3 Department of Chronic Disease Epidemiology, Yale School of Public Health, Yale University, New Haven, CT USA

**Keywords:** DNA methylation quantitative trait loci (mQTL), DNA methylation, Epigenome-wide association analysis (EWAS), Birthweight

## Abstract

**Background:**

Epigenome-wide association studies (EWAS) have helped to define the associations between DNA methylation and many clinicopathologic and developmental traits. Since DNA methylation is affected by genetic variation at certain loci, EWAS associations may be potentially influenced by genetic effects. However, a formal assessment of the value of incorporating genetic variation in EWAS evaluations is lacking especially for multiethnic populations.

**Methods:**

Using single nucleotide polymorphism (SNP) from Illumina Omni Express or Affymetrix PMDA arrays and DNA methylation data from the Illumina 450 K or EPIC array from 1638 newborns of diverse genetic ancestries, we generated DNA methylation quantitative trait loci (mQTL) databases for both array types. We then investigated associations between neonatal DNA methylation and birthweight (incorporating gestational age) using EWAS modeling, and reported how EWAS results were influenced by controlling for mQTLs.

**Results:**

For CpGs on the 450 K array, an average of 15.4% CpGs were assigned as mQTLs, while on the EPIC array, 23.0% CpGs were matched to mQTLs (adjusted *P* value < 0.05). The CpGs associated with SNPs were enriched in the CpG island shore regions. Correcting for mQTLs in the EWAS model for birthweight helped to increase significance levels for top hits. For CpGs overlapping genes associated with birthweight-related pathways (nutrition metabolism, biosynthesis, for example), accounting for mQTLs changed their regression coefficients more dramatically (> 20%) than for other random CpGs.

**Conclusion:**

DNA methylation levels at circa 20% CpGs in the genome were affected by common SNP genotypes. EWAS model fit significantly improved when taking these genetic effects into consideration. Genetic effects were stronger on CpGs overlapping genetic elements associated with control of gene expression.

**Supplementary Information:**

The online version contains supplementary material available at 10.1186/s13148-022-01385-6.

## Background

Epigenome-wide association studies (EWAS) examine the relationship between DNA methylation at individual CpG sites throughout the genome and demographic, environmental, or disease characteristics. DNA methylation is quantified as the fraction of site-specific CpGs that are methylated within a tissue, for example, blood, and typically using Illumina array methodology. While DNA methylation level is a continuous phenotype, its status is primarily a reflection of gene expression control within a particular tissue. DNA methylation may, however, be partially or completely controlled by neighboring genetic factors or polymorphisms, or be itself polymorphic. When these polymorphisms exist within the probes used for measurement, the DNA methylation assay may fail, leading to aberrant data—lists of such CpG sites have been published and these are usually filtered out before the analysis phase [1, 2]. Genetic effects are not limited to such probes, as genetic polymorphisms can have profound *cis* or *trans* impacts on DNA methylation even at a distance—an example would be several of the CpG sites that are known to be strongly impacted by tobacco exposure [3]. Interestingly, some of these are also impacted by SNPs proximal to their sites as *cis* methyl quantitative trait loci [4]. SNPs may both distort the environmental association (e.g., *GFI1* CpG and tobacco) or simply introduce noise without being directly associated with the environment (e.g., *AHRR* and tobacco) [4]. In either case, the lack of accounting for the genetic effect leads to measurement error in associations between environmental and phenotypic characteristics and DNA methylation which should be accounted for in EWAS analysis. Ultimately, both genetic and environmental effects need to be incorporated to understand epigenetic variation associated with environmental, dietary, and other factors.

Birthweight is related to several factors, including gestational age, parity, fetal sex, maternal height, age, and ethnicity [5, 6]. Studies have also shown recurrent DNA methylation variations associated with birthweight corrected for gestational age. Indeed, EWAS studies have established a large set (914 CpG sites) of CpG sites validated over a large meta-analysis of Illumina HM450K data in neonates [7]. Birthweight is also known to have a strong polygenic etiology, with over 430 birthweight-associated SNPs discovered in genome-wide association studies [8, 9]. Here, we consider DNA methylation variation at birth in relation to birthweight variation in a California population of non-Latino White and Latino ethnicities, while accounting for DNA methylation quantitative trait loci (mQTL).

## Results

### Overview of scanned mQTLs in different datasets

**Fig. 1:**
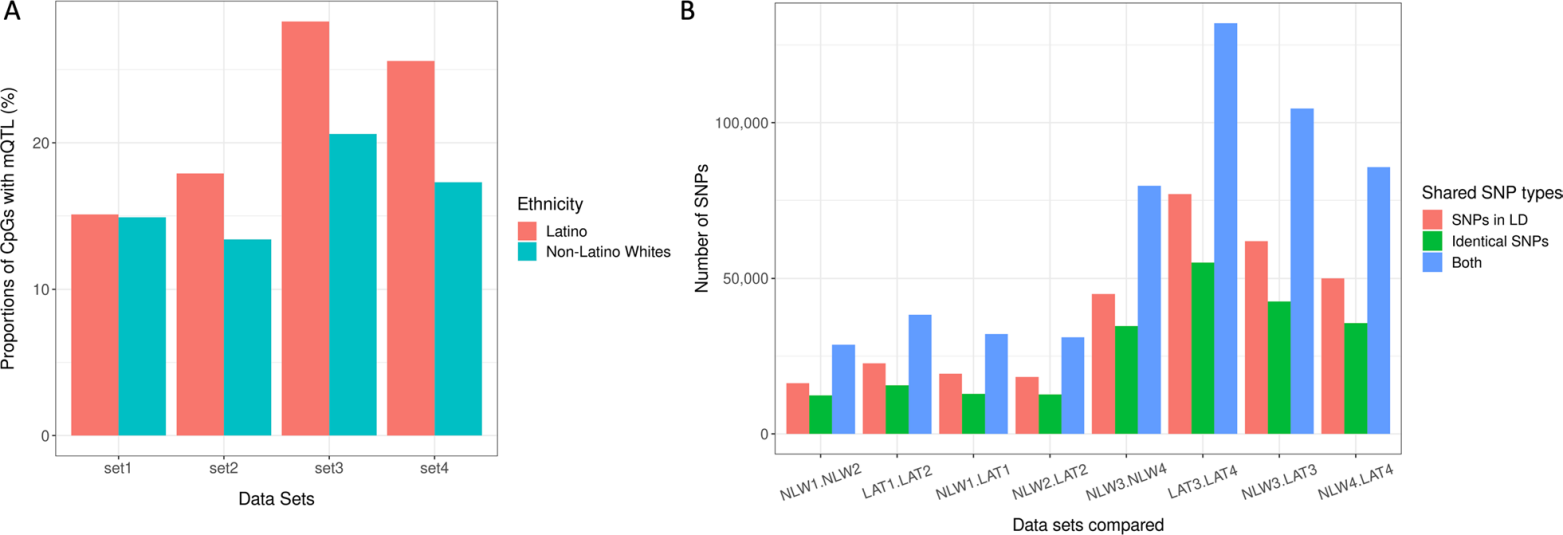
Distributions of scanned mQTLs in different datasets. **A** Proportions of CpGs with a matched significant mQTL in four different datasets by ethnicity. **1** Pair-wise comparisons of datasets by ethnicity on each platform. Compared are number of CpGs with the same SNP assigned as matched mQTLs across datasets, number of CpGs whose mQTLs are in linkage disequilibrium (*R*^2^ > 0.5) across datasets, and number of CpGs whose matched mQTLs fit either of the previous two criteria. *NLW* non-Latino whites; *LAT* Latinos

See Methods section and Additional file 4: Table S1 for a description of datasets and generation of array data used in this study. Briefly, four datasets each containing non-Latino Whites (NLW), and Latinos (LAT) were included. Each dataset has both DNA methylation data (Illumina 450 K or EPIC arrays) and a genome scan for SNPs (Illumina Omni Express or Affymetrix Precision Medicine Diversity Array). See Methods section for scanning and detection of mQTLs in each dataset by ethnicity. For datasets assessed on the 450 K methylation array (Set 1 NLW, Set 1 LAT, Set 2 NLW, and Set 2 LAT), an average of 15.35% CpGs have a matched mQTL at adjusted significance level (*P* < 0.05), while for datasets assessed on the EPIC methylation array (Set 3 NLW, Set 3 LAT, Set 4 NLW, and Set 4 LAT), an average of 22.95% CpGs are matched to mQTLs with adjusted *P* values < 0.05 as its mQTL (Fig. 1A). The larger proportions of mQTLs identified in EPIC arrays are likely due to the larger sample size (*n* = 971) than in sets run on 450 K arrays (*n* = 667). Interestingly, proportions of mQTL-matched CpGs differ by ethnicity. In all four datasets, LAT consistently have a higher proportion of mQTL-matched CpGs comparing to NLW (Fig. 1A), likely due to differences in statistical power. More specifically, sample sizes of LAT subjects were larger than NLW in all four datasets, and CpG-SNP pairs with similar effect sizes could have a higher statistical significance in the LAT cohort because of their larger sample sizes. To investigate if this was the case, we meta-analyzed mQTLs of each ethnicity (NLW: Set 1 NLW, Set 2 NLW, Set 3 NLW, Set 4 NLW; LAT: Set 1 LAT, Set 2 LAT, Set 3 LAT, Set 4 LAT), and for shared CpG-SNP pairs between these ethnicities, we compared effect sizes and *P* values of mQTL effects (Additional file 3: Figure S1). As a result, effect sizes were similar, but *P* values were stronger in Latino subjects, suggesting that the discrepancy was mostly due to sample size differences.

We also examined whether a CpG tends to be paired with the same SNP, or SNPs in linkage disequilibrium (LD) (*R*^2^ > 0.5), across different datasets. We made 8 pair-wise comparisons for datasets assessed on the same DNA methylation platform (Additional file 4: Table S2). For 450 K arrays, SNPs matched to the same CpG across dataset have a higher probability to be in LD with each other than identical. However, for EPIC array, SNPs tend to be identical rather than in LD (Fig. 1B). All overlaps of shared CpG-SNP pairs on either 450 K array or EPIC array datasets are shown in Additional file 3: Figure S2.

### Generation of CpG-mQTL pair databases by DNA methylation arrays

We then combined results from multiple datasets to create an mQTL database for 450 K and EPIC arrays separately, which include CpG-SNP pairs with the strongest associations, which can be used as mQTL covariates in subsequent EWAS analyses. These mQTL databases are used subsequently to account for genetic effects in epigenome-wide association analysis. More specifically, we combined mQTL scanning from Set 1 NLW and LAT, Set 2 NLW and LAT datasets to create a database for the 450 K array, while Set 3 NLW and LAT, Set 4 NLW and LAT datasets were combined to create a database for the EPIC array.

The following combining scheme for each platform was adopted: For each CpG, all SNPs identified as mQTLs in each dataset were recorded, sorted by *P* value, which was computed by meta-analyzing effect sizes from different datasets (between both ethnic groups, and between both datasets) weighted by inverse of standard errors using the “SCHEME STDERR” mode of METAL [10]. As one CpG could be matched to different mQTLs in different datasets, there could be several CpG-SNP pairs for a particular CpG. As a result, on the 450 K array, 243,450 such CpG-SNP pairs were identified for a total of 150,333 CpGs. On the EPIC array, 630,971 CpG-SNP pairs were in the database for a total of 358,325 CpGs.

### Genome-wide distribution of mQTL

We next investigated the location of CpGs with the significant CpG-SNP correlation from each array, and whether they have a higher chance of localizing within regions that play a key biological role.

**Fig. 2:**
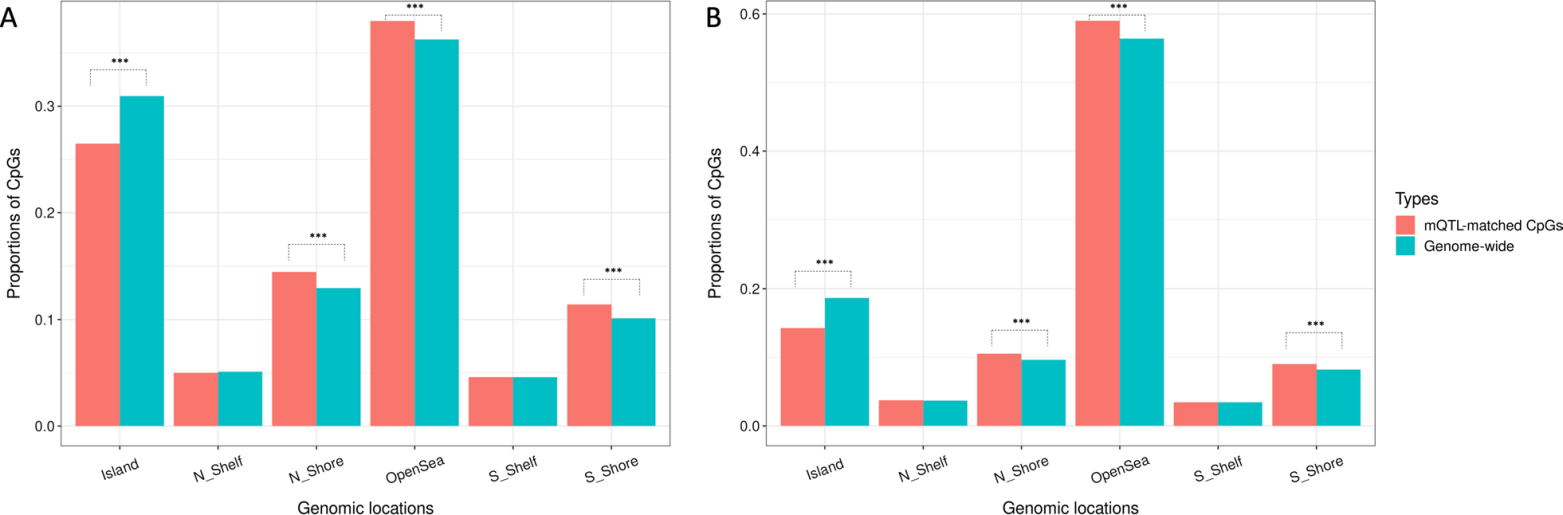
Distributions of different genomic positions of genome-wide CpGs with matched mQTLs. **A** distributions of CpGs on the 450 K methylation array in different regions of the genome. Salmon bars show proportions of CpGs located in each genomic region for CpGs matched to mQTLs, and turquoise bars show distributions for all genome-wide CpGs. Whether these proportions were significantly different were compared using proportion tests (* for *P* < 0.05, ** for *P* < 0.01, and *** for *P* < 0.001). **B** Distributions of CpGs on the EPIC methylation array in different regions of the genome. Similar to (**A**), Salmon bars show proportions of CpGs matched to mQTLs, and turquoise bars show distributions for all CpGs. Proportion tests were done similar to that of (**A**).

On the 450 K array, CpGs with matched mQTLs had a higher probability to be located in northern Shore (N_shore), southern shore (S_shore), and OpenSea regions, comparing to genome-wide CpG distributions. On the EPIC array, mQTL-matched CpGs were less likely to be in the OpenSea region, but enriched in all other regions (Fig. 2A, 2B).

Interestingly, on both arrays, CpGs with matched mQTLs are significantly enriched in CpG island shore regions (either the N_shore or S_shore) comparing to whole-genome distributions (chi-squared test *P* values < 2.2 × 10^–16^ on both arrays) (Additional file 3: Figure S3).

We also conducted enrichment analysis to investigate if CpGs matched to mQTLs are more or less likely to be in regulatory regions. For transcription factors, we gathered all available TFs in the Encyclopedia of DNA Elements (ENCODE) [11] ChiP-seq database for (*N* = 161) in 91 cell types combined; interestingly, 149 were significantly *less* enriched on the 450 K array while no TF site is more enriched. On the EPIC array, 148 were significantly less enriched, while 8 were significantly more enriched. Among the available CpG sites on the arrays we can conclude that TF binding sites are less likely to harbor mQTLs. For histone modification sites, H3K4me3 sites were significantly less enriched for CpGs matched to mQTLs both on 450 K array (fold enrichment = 0.934, *P* value = 1.27 × 10^–80^) and EPIC array (fold change = 0.654, *P* value < 1.00 × 10^–160^). On the contrary, H3K27me3 sites were significantly more enriched for CpGs matched to mQTLs both on 450 K array (fold enrichment = 1.108, *P* value = 1.414 × 10^–85^) and EPIC array (fold change = 1.0395, *P* value = 6.6476 × 10^–22^). Lastly, in terms of enhancer regions for three HSC cell lines, CpG sites in enhancer regions were less enriched for both 450 K and EPIC arrays (450 K array, fold change = 0.895, *P* value = 1.3563 × 10^–122^; EPIC array, fold change = 0.8022, *P* value < 1.00 × 10^–160^).

### Comparison with published *cis* and *trans* mQTL effects

Min et al. [12] reported a database of *in cis* mQTLs using large cohort of subjects of NLW ethnicity. We compared both the effect sizes and *P* values of mQTLs from our databases with Min et al.’s (Additional file 3: Figure S4). In all datasets and in both NLW and LAT populations, there is very low consistency in terms of both effect sizes and *P* values.

It was reported that *in cis* mQTLs have much bigger effect sizes than *in trans* mQTLs [12]. While trans mQTLs should be relevant for our purposes, we were underpowered to capture *trans* mQTL effects. Therefore, we limited our mQTL scanning to *in cis* SNP-CpG pairs. Nevertheless, we still investigated if we could leverage published *trans* mQTL databases to account for *in trans* genetic effects. Recently, studies were published discussing trans-mQTL effects in large cohorts [12, 13]; however, most of these studies focus on subjects of non-Latino white populations, and it is the applicability of *trans*-mQTL databases across ethnicities is not validated.

We aimed to test if *trans*-mQTLs published by Min et al. could be replicated on our Latino and non-Latino White subjects. To achieve this, we extracted 46,148 CpG along with the most significant SNP matched to each CpG from their analysis within our dataset and tested whether the same mQTL effects were present in our dataset. We were able to test 43,464 such CpG-SNP pairs from Set 1 (NLW and LAT), 43,386 from Set 2 (NLW and LAT), 40,247 from Set 3 (NLW and LAT), and 40,360 pairs from Set 4 (NLW and LAT). The replicability of trans-mQTL effects was, however, poor in all NLW and LAT datasets, both in terms of regression coefficients and significance. Additional file 3: Figure S5 shows Set 1 NLW, Set 1 LAT, Set 2 NLW, and Set 2 LAT as examples. We therefore do not consider trans mQTLs effects in the current manuscript.

### EWAS reveals significant CpGs associated with birthweight

Birthweight has been reported to have significant associations with neonatal DNA methylation [7, 14, 15]. However, previous reports did not take into consideration possible effects from genetics. To address this, using the same dataset where mQTL scanning was performed, we conducted an EWAS analysis to investigate the correlation between neonatal DNA methylation and birthweight, while accounting for platform-specific mQTLs. This analysis was performed in all four datasets separately, and meta-analysis was conducted for each array (450 K and EPIC separately). We also conducted the same regression models without accounting for SNPs for comparison.

#### On the 450 K array

In the 450 K array dataset (*n* = 667), using EWAS models accounting for mQTL effects (mQTL-model), we discovered a total of 33 CpGs significantly associated with birthweight after Bonferroni correction, of which 19 CpGs were previously associated with birthweight [7], for example, in the ARID family genes *ARID3A* and *ARID5B* (Additional file 4: Table S3), and 14 CpGs were novel associations. Of the 33 significant CpGs, 15 (45.45%) were corrected for significant mQTLs in the model, and three of these CpGs were located in the transcription start site region of the genes.

We also conducted the same EWAS without accounting for mQTL effects in Sets 1 and 2 (non-mQTL-models), followed by the same meta-analysis like that of models including mQTLs. As a result, none of these hits would have been identified if we did not account for mQTL effects, as only 3 CpGs were significant when mQTLs were not included in the model. This suggested the importance of taking genetic effects into consideration in epigenome-wide association analysis.

**Fig. 3:**
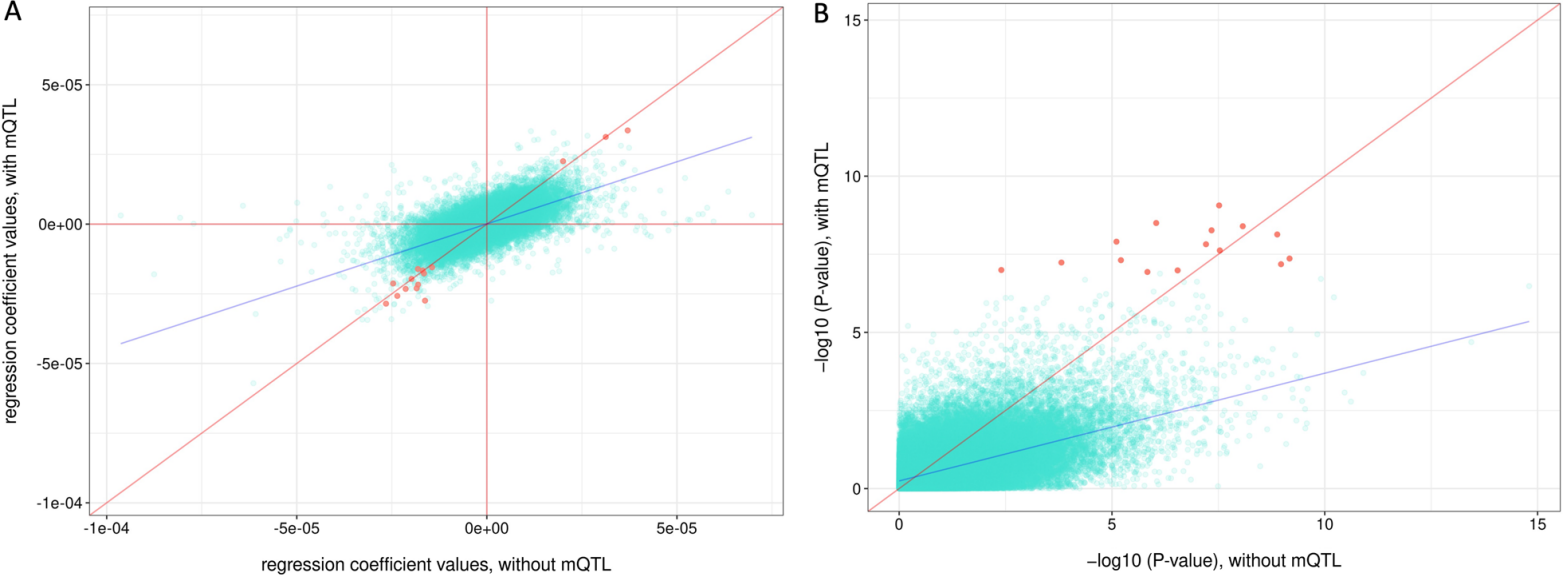
Comparison of birthweight epigenome-wide association analysis results with or without controlling for mQTL (450 K array). Comparison of two EWAS model’s (mQTL-model and non-mQTL-model) results analyzing the correlation between birthweight and DNA methylation for a total of 129,072 CpGs matched to mQTLs. One model included mQTL for each CpG as a covariate (*y*-axis), while the other did not (*x*-axis). For the same CpG, regression effect sizes and *P* values were compared in these two models. **A** regression *β* coefficients from these two models were compared for CpGs on the 450 K array. The 15 CpGs that were significantly associated with birthweight were marked red, and CpGs that were not significant were marked turquoise. Red lines represent *X* = 0, *Y* = 0 and *X* = *Y*, respectively. Blue line represents regression line between *Y* and *X*. **B** Negative log *P* values from these two models were compared, with significant CpGs marked red. Similar to **A**, *X* = *Y* is shown with a red line and blue line shows regression line between *Y* and *X*

We investigated how controlling for mQTL’s genetic effects changed results of our EWAS models. We were able to compare a total of 123,012 CpGs which were matched to mQTLs. Their matched mQTLs’ effects were accounted for in mQTL-models but not in non-mQTL-models. While regression coefficients from EWAS models with or without controlling for mQTLs were in general similar (Fig. 3A), however, inconsistencies were also seen for some CpGs, as for these CpGs, controlling for mQTLs affected EWAS effect sizes to a large extent. However, the regression coefficients for the 15 significant mQTL-matched CpGs seemed to be similar with or without controlling for mQTLs (Fig. 3A).

In terms of *P* values, controlling for genetic effects also affected significance for many CpGs, to a much greater extent than regression coefficients (Fig. 3B). This included the 15 significant mQTL-matched CpGs from the model, and the majority of these significant CpGs became more significant after adjusting for mQTL effects. However, opposite from that of the significant hits, the majority of 450 K CpGs became less significant after adjusting for SNP effects. As shown in Fig. 3B, the majority (80,384 out of 122,997, 65.35%) of non-significant hits (shown in turquoise) were below the *y* = *x* line.

The 450 K results suggest that, in general, controlling for genetic effects can help to identify additional CpG loci that are associated with variation in birthweight.

We also conducted an enrichment test on the 26 genes overlapping significant CpGs identified in the association analysis using Gene Set Enrichment Analysis (GSEA) [16, 17] (Additional file 4: Table S5). “Early-TGFB1 signature” gene set was identified to be strongly enriched (FDR *q* value = 1.92 × 10^–2^), among other sets.

#### On the EPIC array

There has not been a report of large-scale multi-cohort birthweight EWAS on the EPIC array as was performed previously for the 450 K array [7]. In the larger EPIC array sample set (*n* = 971), we identified 3,294 significant CpGs after Bonferroni correction associated with birthweight (Additional file 4: Table S4) from mQTL-model, many of which were not available in the 450 K platform (2112 out of 3294, 64.12%). For example, one of the top hit CpGs cg09797037 in *EXOSC10* (*P* = 8.42 × 10^–31^, direction: + +) is significantly associated with birthweight in our EPIC array data; however, Küpers et al. did not identify this gene in their multi-cohort meta-analysis.

In our EWAS results, 2004 (60.84%) of the significant hits were corrected for mQTL’s genetic effects, at a CpG-SNP mQTL cutoff adjusted *P* value of 0.05. 425 (12.90%) of these CpGs are annotated as transcription starting region (TSS) in the Illumina’s EPIC methylation arrays annotation [18].

Similar to 450 K, we repeated the same EWAS model in both Sets 3 and 4 without controlling for mQTL’s effects (non-mQTL-models). Running the same pipeline (meta-analysis, and multiple correction using Bonferroni), 2216 of significant hits from models including mQTLs as covariates would not have been identified if mQTL effects were not adjusted, accounting for 67.27% of all the significant hits. Similar to that of the 450 K array, this illustrates that adjusting for mQTLs in this EWAS model significantly affected the fundamental landscape of results.

**Fig. 4:**
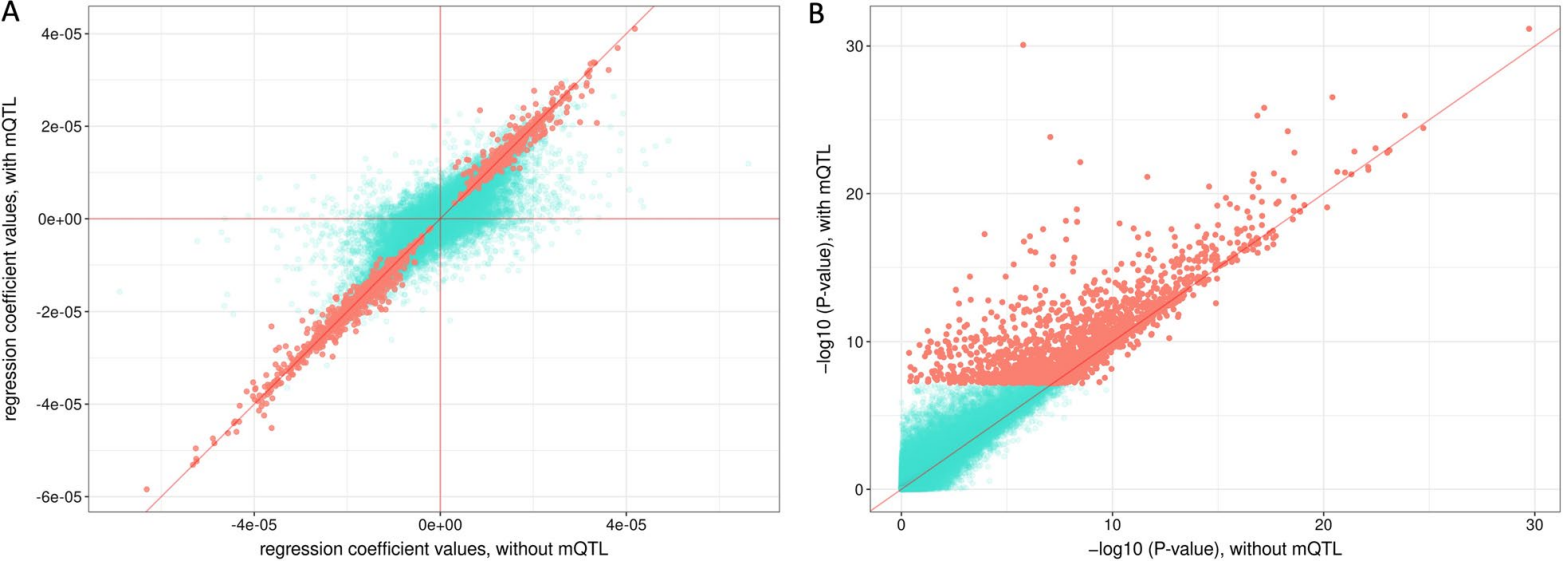
Comparison of birthweight epigenome-wide association analysis results with or without controlling for mQTL (EPIC array). Similar to that of Fig. 3, two EWAS models (mQTL-model and non-mQTL-model) were run. Regression effect sizes and *P* values of 155,852 CpGs were compared. **A** Regression *β* coefficients from these two models were compared for CpGs on the EPIC array. The 3354 CpGs that were significant were marked red, and CpGs that were not significant were marked turquoise. Red lines represent *x* = 0, *y* = 0 and *x* = *y*, respectively. **B** Negative log *P* values from these two models were compared, with significant CpGs marked red. *X* = *y* is shown with a red line

Adding the mQTLs as additional variables also did not alter regression effect sizes appreciably for the 3294 significant CpGs, similarly to the result with the 450 K (Fig. 4A).* P* values were also significantly different after controlling for mQTL, increasing the significance for most significant hits, further suggesting the importance of controlling for genetic effects when conducting EWAS analysis (Fig. 4B). For the majority of all EPIC CpGs, after adjusting for SNP effects, the majority (154,489 out of 294,446, 52.47%) also became more significant (Fig. 4B), opposite from the trend observed on the 450 K array.

We also tested for model fit to test for the effect of mQTL using partial F-tests. For CpGs matched to mQTLs, full model refers to mQTL-model, and reduced model refers to non-mQTL-model (see Methods section for equations of these models). In Set 4, for example, 199,625 out of 296,157 (67.41%) models have a significant partial F-test *P* value. This illustrated for a majority of CpGs, after controlling for matched mQTLs, model fit significantly improved.

GSEA was also performed using top 50 genes from the EWAS results similar to that of 450 K (Additional file 4: Table S6). Enriched gene sets include tissue maturation (FDR* q* value = 3.87 × 10^–04^) and abnormal inflammatory response (FDR q value = 3.87 × 10^–04^).

#### Shared CpGs

For shared, or in-common CpGs (CpGs that are on both 450 K and EPIC array), we controlled for mQTLs for in-common CpGs described in the method section for each dataset and meta-analyzed all four datasets. Results were similar: controlling for mQTL effects seemed to increase significance of birthweight effects (Additional file 3: Figure S6).

### Investigating CpGs whose regression coefficients were significantly affected by mQTLs

#### On the 450 K array

We found that after correcting for mQTLs, regression coefficients of some CpGs changed more significantly than others, suggesting that the association between these CpGs and birthweight was more heavily distorted by mQTLs. Taking genetic effects into consideration is of vital importance for this group of CpGs.

Among all the significant CpGs in the association analysis after FDR correction, there are in total 93 CpGs whose effect sizes changed more than 20% in either direction after correcting for mQTL effects. Pathway analyses suggest these CpGs are enriched in cell proliferation and energy related pathways. For example, KEGG of these CpGs showed top pathways include glycerophospholipid metabolism (KEGG ID 00,564, adjusted *P* value = 1.08 × 10^–80^) and GnRH signaling pathway (KEGG ID 004,912, adjusted *P* value = 1.08 × 10^–80^) among top pathways (Additional file 4: Table S7).

#### On the EPIC array

The EPIC array was able to identify 1711 CpGs with significantly changed effect sizes after correcting for mQTL, using the same filtering as that of 450 K. Pathway analyses also revealed that these CpGs are enriched in energy and metabolism functions. In GO pathway analysis, ATPase regulator activity (GO ID 0060590, adjusted *P* value = 3.20 × 10^–08^) and chemokine activity (GO ID 0045600, adjusted *P* value = 0.044) are among significantly enriched pathways (Additional file 4: Table S8).

These results suggested that, for CpGs overlapping with genes in pathways associated with birthweight, including biosynthesis, metabolism, and leukemia (high birthweight was a risk factor for leukemia [19]), their association analyses with birthweight were distorted heavily by genetic effects.

### Correcting for maternal weight gain as an additional covariate did not significantly change EWAS results

It has been reported before that excessive maternal gain was associated with birthweight [20–22]. Therefore, maternal weight gain during pregnancy could also influence birthweight EWAS models. As a sensitivity analysis, we controlled for maternal weight gain as an additional covariate. As a result, neither *P* values nor regression coefficients were significantly altered. (Set 4 results were shown as an example in Additional file 3: Figure S7.)

## Discussion

While epigenome-wide analyses (EWAS) have helped identify the correlation between DNA methylation and key clinical or biological traits, the effects of genetic factors are generally ignored in such studies. Using four multi-ethnic datasets, we generated SNP-CpG databases that could help us understand the role played by mQTLs in EWAS studies using a well-studied covariate that is related to DNA methylation at birth (birthweight), for both 450 K and EPIC arrays separately.

Several studies [12, 13] have explored mQTL effects in large cohorts, mostly of European ancestry. However, populations of varied ethnicities might have different SNP-CpG interactions. For example, the Latino population is recently admixed European, Amerindian, and African ancestries, and mQTL features can be inherited from all three ancestral groups. By adjusting for ancestral-variant principle components (EPISTRUCTURE), we aimed to account for such heterogeneity on a global scale. Specific mQTLs on a local scale may not be adequately controlled with global principal components, necessitating local adjustments. We attempted to use published mQTLs for this purpose; however, replicability between databases seems to be poor, both *in cis* and *in trans* in our data*.* We tested consistency of mQTL effect sizes and significance from our datasets with Min et al., and in all datasets in both NLW or LAT populations, there is very low consistency. Reasons for low replicability are unclear, but may relate to differences in technical batch effects, SNP array designs, study population age structure, and systematic differences from population structures. Either way, this suggests in order to account for genetic effects in EWAS models, leveraging published mQTL databases might be less ideal than doing dataset-specific mQTL scanning particularly when population substructure varies between intended training and test datasets.

We assessed DNA methylation using two different Illumina arrays platforms (450 K and EPIC). Since the EPIC array includes a majority of 450 K array probes, we conducted some analyses on shared probes between both arrays using all datasets. However, scanning mQTLs and applying EWAS models on separate array types are justified, because batch effects in sample processing and data normalization could bias results dramatically. Furthermore, 450 K and EPIC arrays had differences in design even for some of the same CpGs, and it has been previously reported that Type I probes had lower individual site correlations than Type II probes [23].

Nevertheless, on both arrays those CpGs that matched to mQTLs were more likely to be in CpG island shore regions (N_shore or S_shore) compared to other annotated regions. CpG island shores are 2 kb regions flanking a CpG island [24], and DNA methylation in the shore regions has been reported to be associated with both disease traits such as Alzheimer’s disease [25] and chronic lymphocytic leukemia [26], as well as negatively associated with gene expression levels [24, 27]. We reported that methylation of CpGs in the shore regions was also more likely to be affected by genotypes, suggesting that previously reported shore methylation and gene expression associations were likely in fact expression quantitative trait loci (eQTL) effects and key markers of population trait variability. In fact, of all the 596,249 SNPs that were matched to a CpG in our results, 325,556 (54.60%) were significant eQTLs for the whole blood tissue based on the Genotype-Tissue Expression (GTEx) data. Furthermore, in general, gene control regions (including transcription binding sites, histone modification sites, enhancer regions, etc.) tend to eschew mQTLs which may indicate negative evolutionary selection for mQTLs. In other words, the genetic regulatory motifs are unlikely to tolerate polymorphic variability in DNA methylation levels of nearby sequences. However, polycomb repression-associated H3K27me3 sites were an interesting exception. They preferentially favored CpGs with mQTLs when compared to the remainder of the array.

Incorporating this mQTL database, we performed EWAS analysis investigating the association between birthweight and neonatal DNA methylation. While this relationship has been reported by multi-cohort meta-analysis [7], genetic effects were not previously taken into consideration. We identified some similar hits as before, for example, at *ARID3A* and *ARID5B*. The AT‑rich interacting domain (ARID) family proteins bind to DNA [28] and play roles in transcriptional regulation during cell proliferation, differentiation, and development [29]. Other top genes overlapping with significant CpGs include *PLD2* (cancer development and progression [30, 31]), and *TGFB2* (regulation of angiogenesis and heart development [32, 33]). We were also able to identify CpGs located in genes not previously reported in EWAS studies.

The EPIC array contains almost twice as many probes as the 450 K array, and we were able to identify other genes, on top of 450 K results, significantly related to birthweight. For example, *EXOSC10* was involved in ATP/ITP metabolism pathway. Gusev et al. reported that its expression level was associated with birthweight in a TWAS study [34]. Some other notable genes include *IL21R* (interleukin 21 receptor, transducing the growth promoting signal of IL21 [35]), *IPO9* (associated with waist circumference [36], fat-free mass or lean body mass [37], and body mass index [38] in previous GWAS studies), and *ST6GALNAC4* (catalyzing the transfer of sialic acid from CMP-sialic acid to galactose-containing substrates [39]).

We noticed that after adjusting for mQTLs, the majority of 450 K array CpGs became less significant, but this trend was reversed on the EPIC array. An explanation for this difference was not immediately clear to us. We hypothesized that this was mostly due to array and sample size differences. The same probe can have different designs on 450 K and EPIC arrays, and this discrepancy could contribute to such differences. Additionally, sample size was larger for EPIC array datasets (*N* = 971) than 450 K array (*N* = 667), allowing the regression model to have better and more stable results. In fact, when we looked at probes on both 450 K and EPIC arrays from all our datasets (Additional file 3: Figure S6B), the behavior became very similar to EPIC array results. Therefore, we expect when sample size is large and DNA methylation is captured more accurately; in general, CpGs will become more significant after mQTL corrections.

Interestingly, adjusting for mQTLs had a larger effect on regression coefficient values for a group of CpGs compared to others, most regression coefficients (67%) increasing. To understand the characteristics for these CpGs, we performed pathway analyses and found that they were highly enriched for energy metabolism and biological synthesis pathways. This suggested that for CpGs overlapping genes strongly connected to the outcome (birthweight in our study) in EWAS analyses, accounting for genetic effects was especially important.

We note that it is appropriate to control for mQTL effects in EWAS models when DNA methylation is the outcome in the causal pathway. In other situations, for example, when DNA methylation is an instrumental variable of another causal factor that leads to the phenotype variation, the benefit of controlling for mQTL effects is complicated by additional considerations, which will require additional model specifications. Indeed, the “top hits” from any mQTL-adjusted models should be checked to assure that correct model assumptions are met.

There are some drawbacks of our study. We only had access to DNA methylation array data, instead of whole-genome bisulfite sequencing data, which could limit our ability to detect other key SNP-CpG loci. Moreover, although our datasets contain multi-ethnic subjects, we only had access to significant numbers of NLW and LAT subjects and have ignored other ethnic groups. Once data are available, it will be of interest to investigate how our findings might change in other ethnic groups including African-Americans and Asians.

In summary, it is of value to account for genetic effects when performing EWAS models. Our matrix of SNP-CpG methylation effects presented in the current analysis can be used directly for non-Latino White and Latino populations on both types of Illumina DNA methylation array data. This will allow accounting for genetic bias, especially for CpGs overlapping key genes, and potentially help to identify more associations that were not possible to detect without concurrent adjustment for mQTL effects.

## Methods

### Study subjects

Four separate datasets are used in this analysis (Additional file 4: Table S1). Three datasets (Set 1, 168 non-Latino Whites (NLW) and 174 Latinos (LAT), Set 2, 105 NLW and 220 LAT, and Set 3, 137 NLW and 356 LAT) are from California Childhood Leukemia Study (CCLS) project which involved active recruitment of children with leukemia and healthy controls throughout California between 1995 and 2015 [40]. A separate sample set (Set 4, 160 NLW and 318 LAT) was derived independently from a separate leukemia case–control sample set with subjects born within a five-county Southern California region as described [41]. As these 4 sets were designed and created at separate times, we analyzed them separately without pooling individual level data. Our analyses were limited to self-reported LAT and NLW due to a lack of power for the analysis of other population groups.

This study was approved by the State of California Committee for the Protection of Human Subjects, and the University of Southern California and University of California, Berkeley institutional review boards.

### Genome-wide DNA methylation arrays

DNA was extracted from 1/3 of each newborn dried blood spots (DBS) (~ 1.4 cm diameter) using the Qiagen DNA Investigator blood card protocol, and bisulfite conversion performed using Zymo EZ DNA Methylation kits. Bisulfite-converted DNA samples were randomized.

Sets 1 and 2 were assayed on Illumina Infinium Methylation450K Beadchip genome-wide DNA methylation arrays (referred to as 450 K array from here); Sets 3 and 4 were assayed on Illumina Infinium Methylation850K Beadchip genome-wide EPIC methylation arrays (referred to as EPIC array from here).

### Genome-wide SNP genotype arrays

Additional DNA was isolated from DBS for genotyping. Sets 1, 2, 3 were genotyped on Illumina Omni Express arrays as previously described [42], while Set 4 was genotyped on the Affymetrix Precision Medicine Diversity Array (PMDA) array.

### Genotype data preprocessing

Pre-imputation quality control (QC) was done in Sets 1, 2, 3 and Set 4 separately. Genotyped variants as well as subjects with missing call rates exceeding 5% were excluded. Variants with Hardy–Weinberg equilibrium *P* value < 10^–4^ or with a minor allele frequency < 0.01 were also excluded. We then imputed SNP data on the TOPMed imputation server using TOPMed Imputation Reference panel consisting of 97,256 samples with high-quality whole-genome sequencing data as reference population [43–45], and after imputation, for Sets 1, 2, 4 and Set 3 separately, we included imputed SNPs with an R^2^ score higher than 0.6 and excluded SNPs with a minor allele frequency (MAF) < 0.01. Lastly, since Sets 3 and 4 were measured on different DNA methylation arrays, Set 3 and Set 4 genetic data were harmonized and combined by taking only the shared SNPs from each set. This resulted in 9,129,560 SNPs for Set 1, 9,240,323 SNPs for Set 2, and 8,974,195 SNPs for sets 3 and 4.

### DNA methylation array data preprocessing and annotation

DNA methylation data were normalized using R package “Minfi,” and “funnorm” normalization [46] was performed for each dataset with noob background correction, followed by BMIQ normalization [47]. CpG probes and subjects with more than 5% missingness were removed from normalized data. The remaining missing values were imputed using “impute” package as described in our previous publication [48]. For association analysis, probes located on sex chromosomes as well as CpGs or probes that overlap with SNP sites with MAF > 5% were excluded.

### Scanning of genome-wide mQTLs for each CpG site

Scanning of mQTLs *in cis* was done using QTLtools [49] in each dataset stratified by self-reported ancestry (NLW and LAT) using the parameters described below. This created eight datasets for mQTL scanning: Set 1 NLW, Set 1 LAT, Set 2 NLW, Set 2 LAT, Set 3 NLW, Set 3 LAT, Set 4 NLW, and Set 4 LAT.

We did a permutation test (*n* = 1000) for each CpG and SNPs located within a 2 million base pair window flanking that CpG, the default setting for QTLtools. This analysis outputs the top *in cis* SNP associated with each CpG for each dataset by ancestry. Covariates controlled for in this mQTL screening analysis included sex, batch effect at the time of DNA methylation measurement, and first 5 genetic PCs to account for remaining heterogeneity within ancestry groups. CpG-SNP pairs from each dataset with an association adjusted *P* value < 0.05 were included in downstream analysis. Multiple testing adjustment was based on the number of variants and phenotypes tested in *cis* given by the fitted beta distribution as described in Delaneau [49]. Briefly, null distribution was empirically characterized through permutation (*n* = 1000 in our study), and adjusted *P* values were computed by assessing how likely the nominal* P* value was from this null distribution.

To obtain a harmonized mQTL database for each DNA methylation array type (450 K and EPIC, in Additional files 1, 2), we meta-analyzed outputs from datasets by platform, and for each CpG, we selected the SNP with the most significant *P* value as the mQTL for that CpG. More specifically, 450 K mQTLs were based on results of meta-analysis of Set 1 NLW, Set 1 LAT, Set 2 NLW, and Set 2 LAT, while EPIC mQTLs were based on results of meta-analysis of Set 3 NLW, Set 3 LAT, Set 4 NLW, and Set 4 LAT.

### Enrichment analysis

To investigate the potential functional significance of genetic influence on CpG sites, we compared CpGs with QTLs to those without across the whole array with regards to genomic functional annotations. A significant increase or decrease in overlap with transcription factor (TF) binding sites, histone modification markers (H3K4me3 and H3K27me3), and previously identified enhancer regions for three HSC cell lines may provide insight into functional relevance to mQTL polymorphic variation in blood. The number of SNP-matched CpGs and array-wide CpGs overlapping each feature was evaluated by the Fisher’s exact test.

TF binding sites for 161 transcription factors in 91 cell types combined were downloaded from the ENCODE project (wgEncodeRegTfbsClusteredV3.bed). Histone modification data were downloaded for cell line E035, a primary HSC, from the Roadmap Epigenomics Mapping Consortium database [50]. Enhancer sites for HSC cell lines (BI_CD34_Primary_RO01536, BI_CD34_Primary_RO01480, and BI_CD34_Primary_RO01549) were acquired from a previously published study [51].

### Assessment and adjustment of cell-type heterogeneity

Reference-based deconvolution of blood cell proportions was obtained using the Identifying Optimal Libraries (IDOL) algorithm from* R* package “FlowSorted.Blood.EPIC.” Reference cord blood data were derived from* R* package “FlowSorted.CordBloodCombined.450 k” [52]. We were able to deconvolute proportions of monocytes, granulocytes, natural killer cells, B lymphocytes, CD4 T lymphocytes, CD8 T lymphocytes, and nucleated red blood cells, which were later used to correct for cell-type heterogeneity in the regression analysis.

### Epigenome-wide association analyses

For each CpG, a linear regression model (mQTL models) was fit using methylation β value as the dependent variable. Independent variables include assigned mQTL if there is one, birthweight (the major variable of interest in this study), batch variable, sex, case control status of childhood acute lymphoblastic leukemia (ALL) as a selection variable (to avoid possible bias effect from ALL since some of our subjects were diagnosed with ALL), gestational age, 10 genetic PCs for ancestry heterogeneity, and deconvoluted cell proportions (excluding granulocytes to avoid collinearity) for cell-type heterogeneity. The equation for mQTL-models is:

$$\begin{aligned} & \text{Methylation}\; \beta \; \text{value} \sim \text{birth} \;\text{weight} \left( \text{gram} \right) + \text{sex} + \text{gestational}\; \text{age} \left( \text{weeks} \right) + \text{Batch} \\ & + \text{selection}\; \text{factor} \left( {\text{case} \text{control} \;\text{status}\; \text{of} \;\text{ALL}} \right) + \text{CD8T} + \text{CD4T} + \text{NK} \\ & + \text{Bcell} + \text{Mono} + \text{nRBC} + \text{PC}1 + \cdots + \text{PC}10 + \text{mQTL} \left( {\text{if}\; \text{present}} \right) \\ \end{aligned}$$

Models without controlling for mQTL effects (non-mQTL models) were also performed to compare results. The equation for non-mQTL-models is:

$$\begin{aligned} & \text{Methylation}\; \beta \;\text{value} \sim \text{birth} \;\text{weight} \left( \text{gram} \right) + \text{sex} + \text{gestational}\; \text{age} \left( \text{weeks} \right) + \text{Batch} \\ & + \text{selection}\; \text{factor} \left( {\text{case}\; \text{control}\; \text{status} \;\text{of}\; \text{ALL}} \right) + \text{CD8T} + \text{CD4T} + \text{NK} \\ & + \text{Bcell} + \text{Mono} + \text{nRBC} + \text{PC}1 + \cdots + \text{PC}10 \\ \end{aligned}$$

CpGs were excluded if they overlap with SNPs with more than 5% MAF, on chromosomes X or Y, or with 50% missing values. Such model was performed in all four datasets (Set 1, Set 2, Set 3, and Set 4), and the results were meta-analyzed by platform (450 K array and EPIC array). Subjects with gestational age shorter than 30 weeks (*n* = 9) were excluded from this analysis.

Sensitivity analyses were run by including maternal weight gain during pregnancy as an additional covariate. The equation for sensitivity models is:

$$\begin{aligned} & \text{Methylation}\; \beta \;\text{value} \sim \text{birth} \;\text{weight} \left( \text{gram} \right) + \text{sex} + \text{gestational} \;\text{age} \left( \text{weeks} \right) + \text{Batch} \\ & + \text{selection} \;\text{factor} \left( \text{case} \text{control}\; \text{status} \;\text{of}\; \text{ALL} \right) + \text{CD8T} + \text{CD4T} + \text{NK} \\ & + \text{Bcell} + \text{Mono} + \text{nRBC} + \text{PC}1 + \cdots + \text{PC}10 + \text{maternal} \;\text{weight} \;\text{gain} \\ & + \text{mQTL} \left( {\text{if}\; \text{present}} \right) \\ \end{aligned}$$

To maximize power to discover mQTLs for CpGs that are on both 450 K and EPIC arrays (common CpGs), we meta-analyzed all 8 datasets, from both array platforms. This created another mQTL dataset for in-common CpGs only. We also conducted separate birthweight EWAS model for in-common CpGs only, controlling for the mQTLs for these CpGs. Bonferroni correction was then done to adjust for multiple testing, with adjusted* P* value < 0.05 as threshold for statistical significance.

## Supplementary Information

**Additional file 1.** mQTL database for 450K methylation array.

**Additional file 2.** mQTL database for EPIC methylation array.

**Additional file 3.** Supplement Figures S1–S7.

**Additional file 4.** Supplement Tables S1–S8.

## Data Availability

Our data are derived from the California Biobank. We respectfully are unable to share raw, individual genetic data freely with other investigators. Should we be contacted by other investigators who would like to use the data; we will direct them to the California Department of Public Health Institutional Review Board to establish their own approved protocol to utilize the data, which can then be shared peer-to-peer.
