# Supplementary material for: Incorporation of DNA methylation quantitative trait loci (mQTLs) in epigenome-wide association analysis: application to birthweight effects in neonatal whole blood
Source: Clin Epigenetics. 2022 Dec 1;14:158. doi: 10.1186/s13148-022-01385-6 (PMC9714153; doi:10.1186/s13148-022-01385-6)

**Supplement figures**

**Figure S1 Comparison of mQTL effects in NLW and LAT populations**

mQTL results of all datasets (Sets 1,2,3,4) of the same ancestry were meta-analyzed to compare the CpG-SNP associations in different ancestry groups, with SNPs coded by their minor allele frequency (MAF). **S1A.** comparison of mQTL effect sies in NLW population (X-axis) and LAT population (Y-axis). **S1B.** comparison of log P values of mQTL effects in NLW population (X-axis) and LAT population (Y-axis).

**
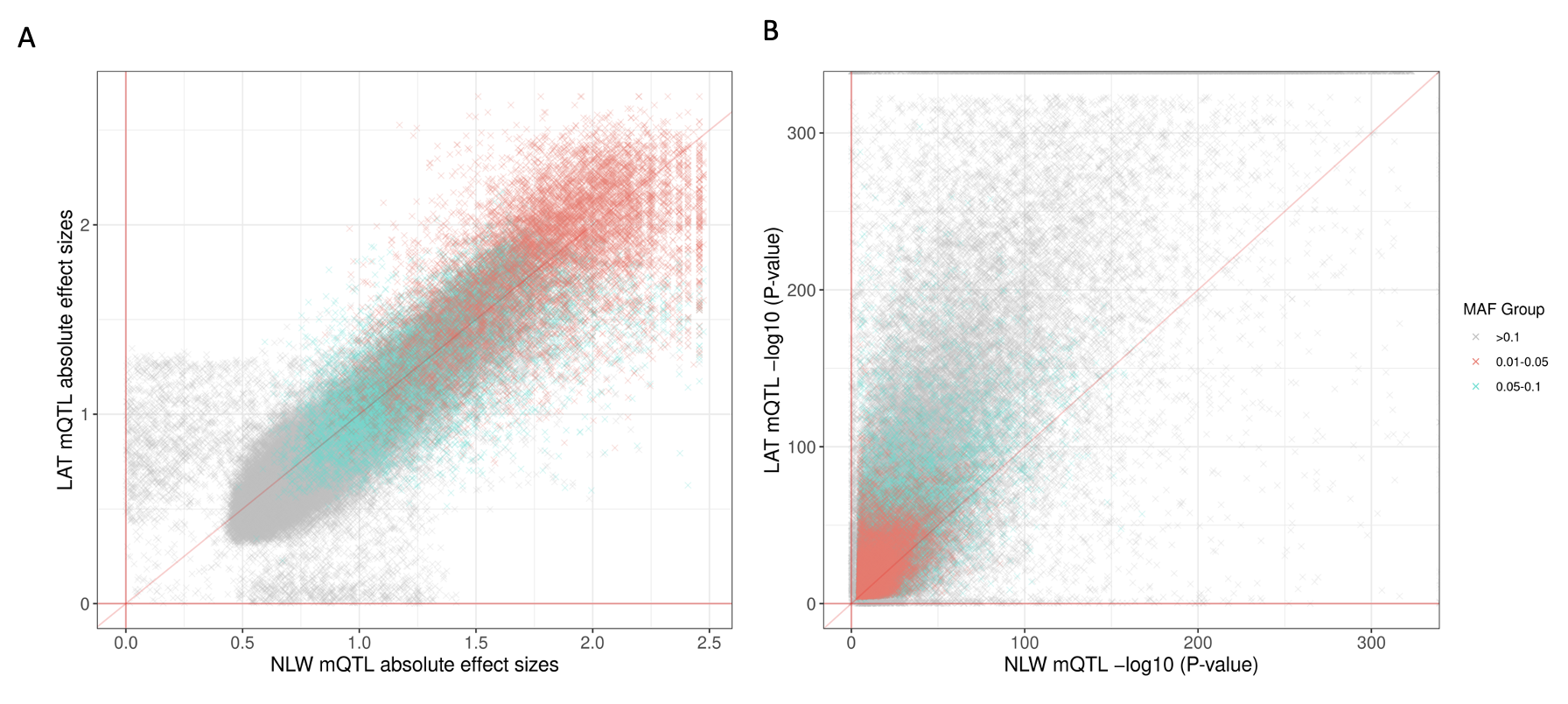
**

**Figure S2 Number and overlap of scanned mQTLs in all four datasets**

CpG DNA methylation levels for Set 1 and Set 2 were measured on 450K arrays, and for Set 3 and Set 4 on EPIC arrays. They were scanned for one SNP (cis mQTL) most significantly associated with each CpG, by different ancestries (non-Latino White and Latino). Figure shows the exact overlap of these CpG-SNP pairs across different datasets and/or ancestries. **S2A**. overlap of scanned CpG-SNP pairs (CpG and its matched mQTL) of datasets with methylation measured on 450K array (Set 1 and Set 2). **S2B**. overlap of scanned CpG-SNP pairs (CpG and its matched mQTL) of dataset 3 and 4, with methylation measured on EPIC array.


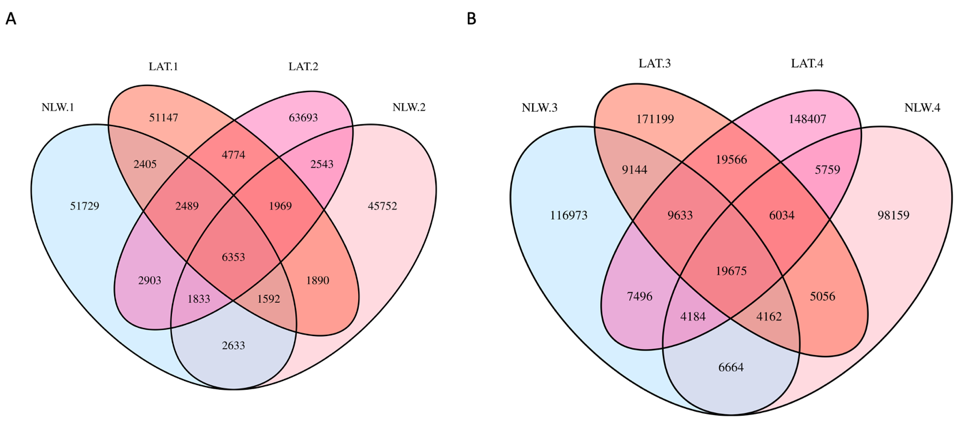


**Figure S3 Distribution of CpGs with matched mQTLs, for shared CpGs only, using all 8 datasets.**

**
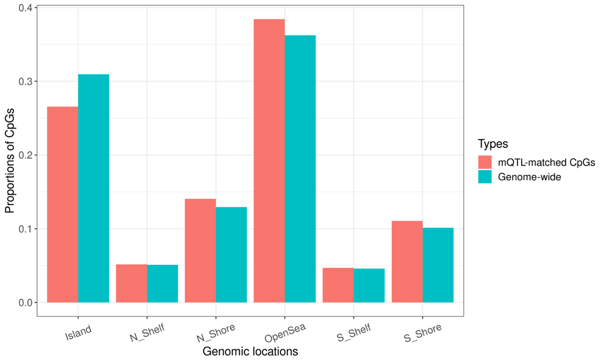
**

**Figure S4 Comparisons of *cis* mQTL effect sizes and log P values between CCLS datasets and *cis* mQTL database from Min et al**

**S4A.** Left panel, Comparison of the effect sizes of *in cis* mQTLs between Set 1 NLW (X axis) and Min et al (Y axis) (Pearson correlation coefficient 0.45). Right panel, Comparison of the log P values of *in cis* mQTLs between Set 1 NLW (X axis) and Min et al (Y axis) (Pearson correlation coefficient 0.12). Y=X is drawn with a red line. **S4B,** similar to that of S4A for Set 1 LAT subjects. (correlation for effect sizes 0.44, for P values 0.12). **S4C,** Set 2 NLW subjects (correlation for effect sizes 0.43, for P values 0.10). **S4D**, Set 2 LAT subjects (correlation for effect sizes 0.45, for P values 0.14). **S4E,** Set 3 NLW subjects (correlation for effect sizes 0.44, for P values 0.13). **S4F**, Set 3 LAT subjects (correlation for effect sizes 0.45, for P values 0.18). **S4G,** Set 4 NLW subjects (correlation for effect sizes 0.43, for P values 0.12). **S4H**, Set 4 LAT subjects (correlation for effect sizes 0.45, for P values 0.18).


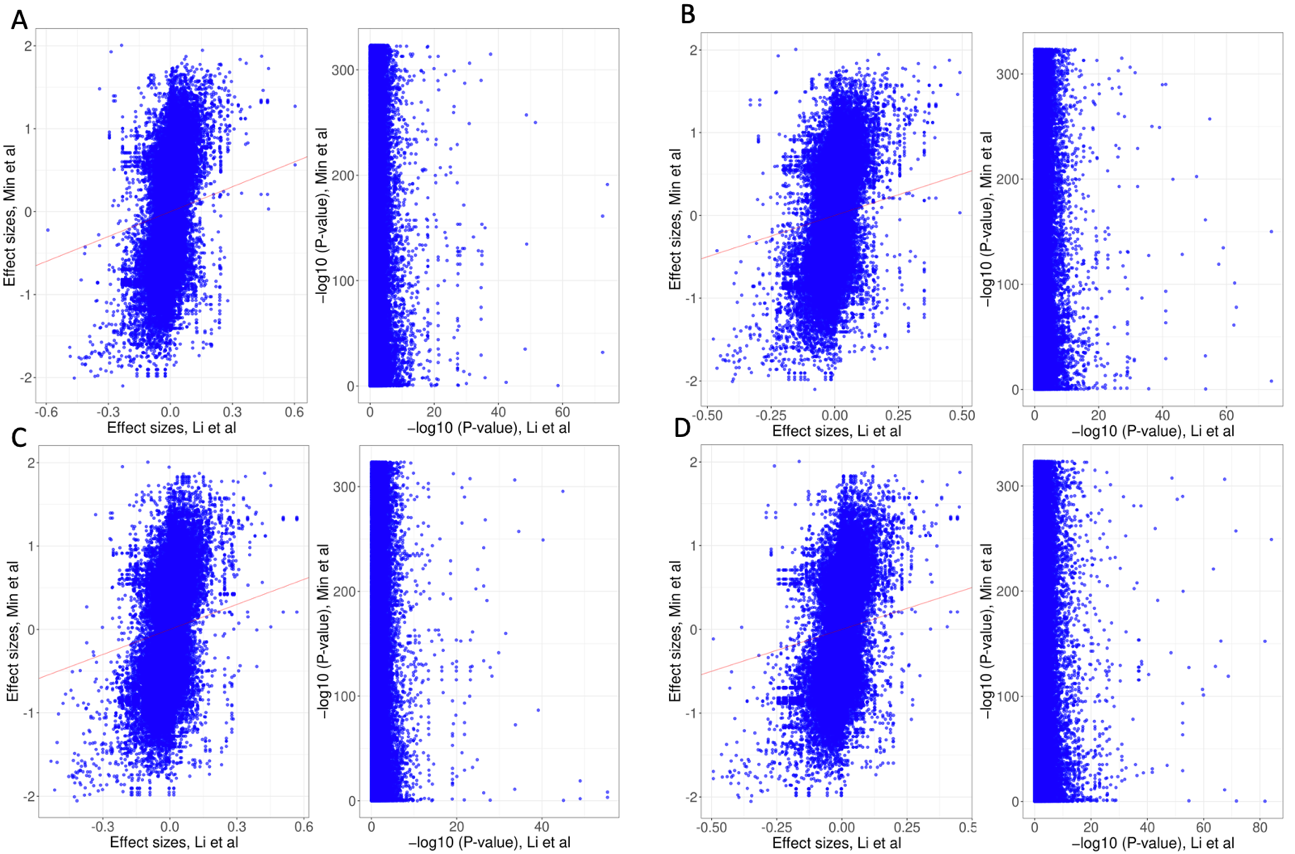

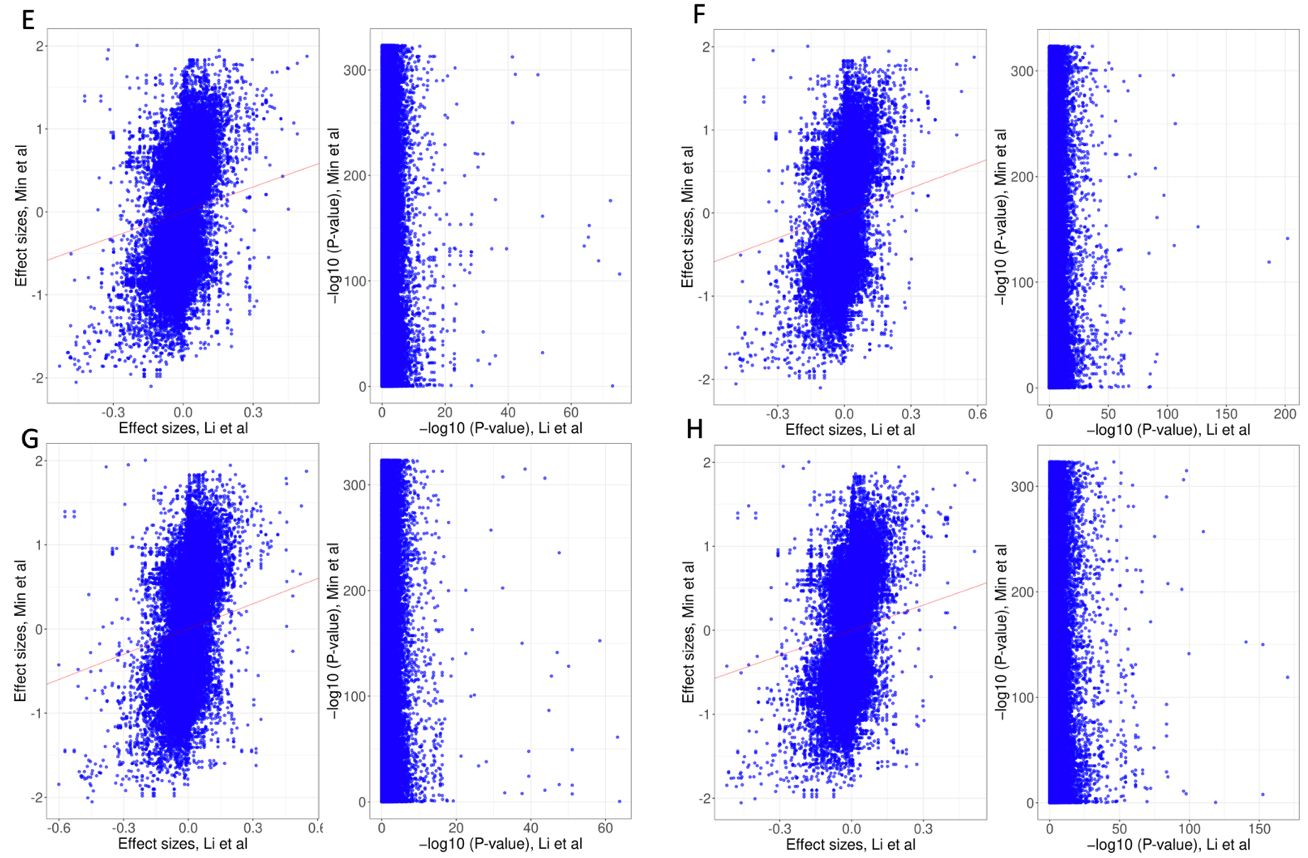


**Figure S5 Comparisons of trans mQTL effect sizes and log P values between CCLS datasets and trans mQTL database from Min et al,**

**S5A.** Left panel, Comparison of the effect sizes of *in trans* mQTLs between Set 1 NLW (X axis) and Min et al (Y axis). Right panel, Comparison of the log P values of *in trans* mQTLs between Set 1 NLW (X axis) and Min et al (Y axis) (correlation for effect sizes 0.35, for P values 0.10). Y=X is drawn with a red line. **S5B,** similar to that of S4A for Set 1 LAT subjects (correlation for effect sizes 0.32, for P values 0.09). **S5C,** Set 2 NLW subjects (correlation for effect sizes 0.34, for P values 0.09). **S5D**, Set 2 LAT subjects (correlation for effect sizes 0.38, for P values 0.09). **S5E,** Set 3 NLW subjects (correlation for effect sizes 0.37, for P values 0.11). **S5F**, Set 3 LAT subjects (correlation for effect sizes 0.41, for P values 0.19). **S5G,** Set 4 NLW subjects (correlation for effect sizes 0.32, for P values 0.10). **S5H**, Set 4 LAT subjects (correlation for effect sizes 0.40, for P values 0.18).


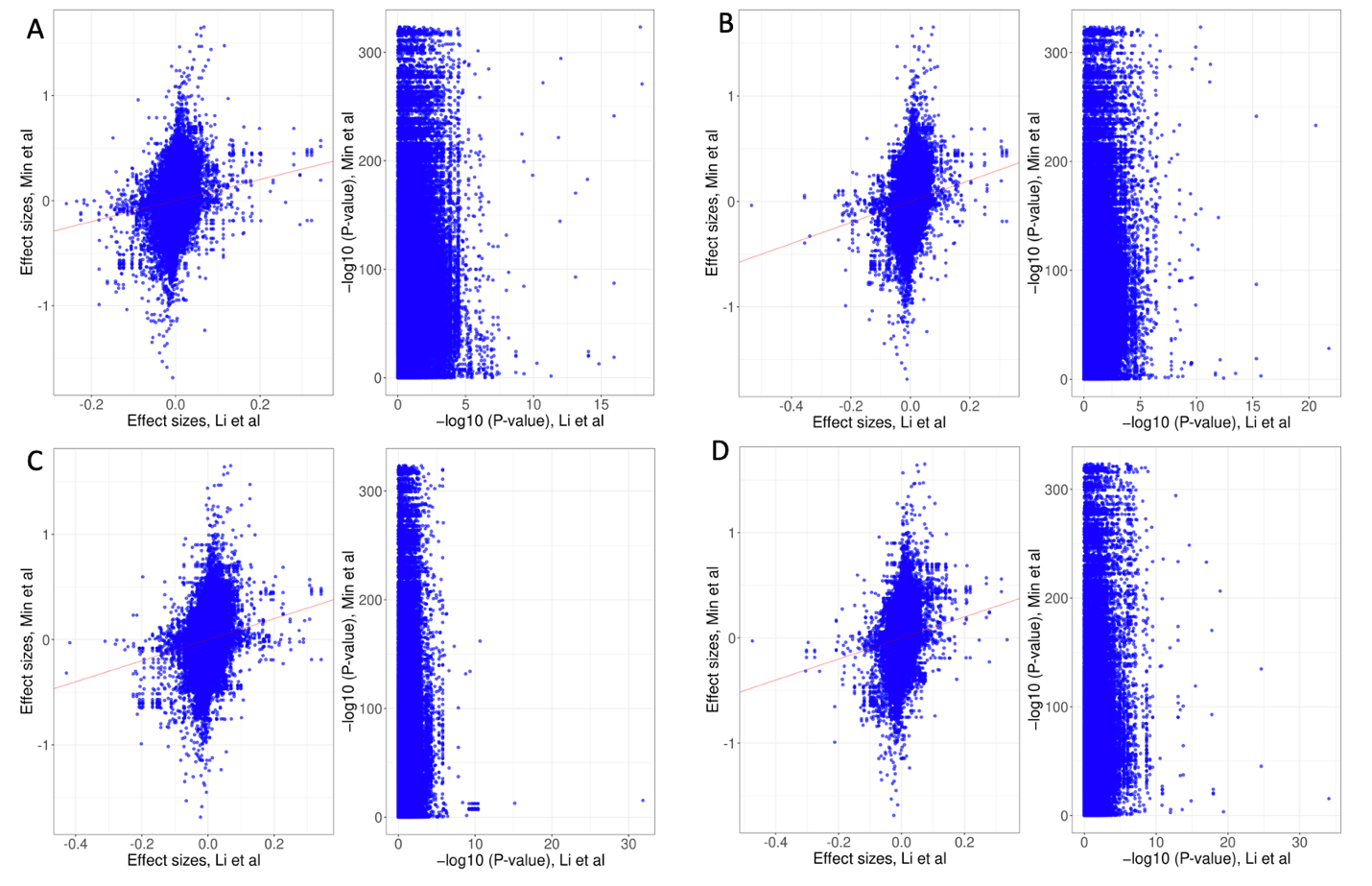

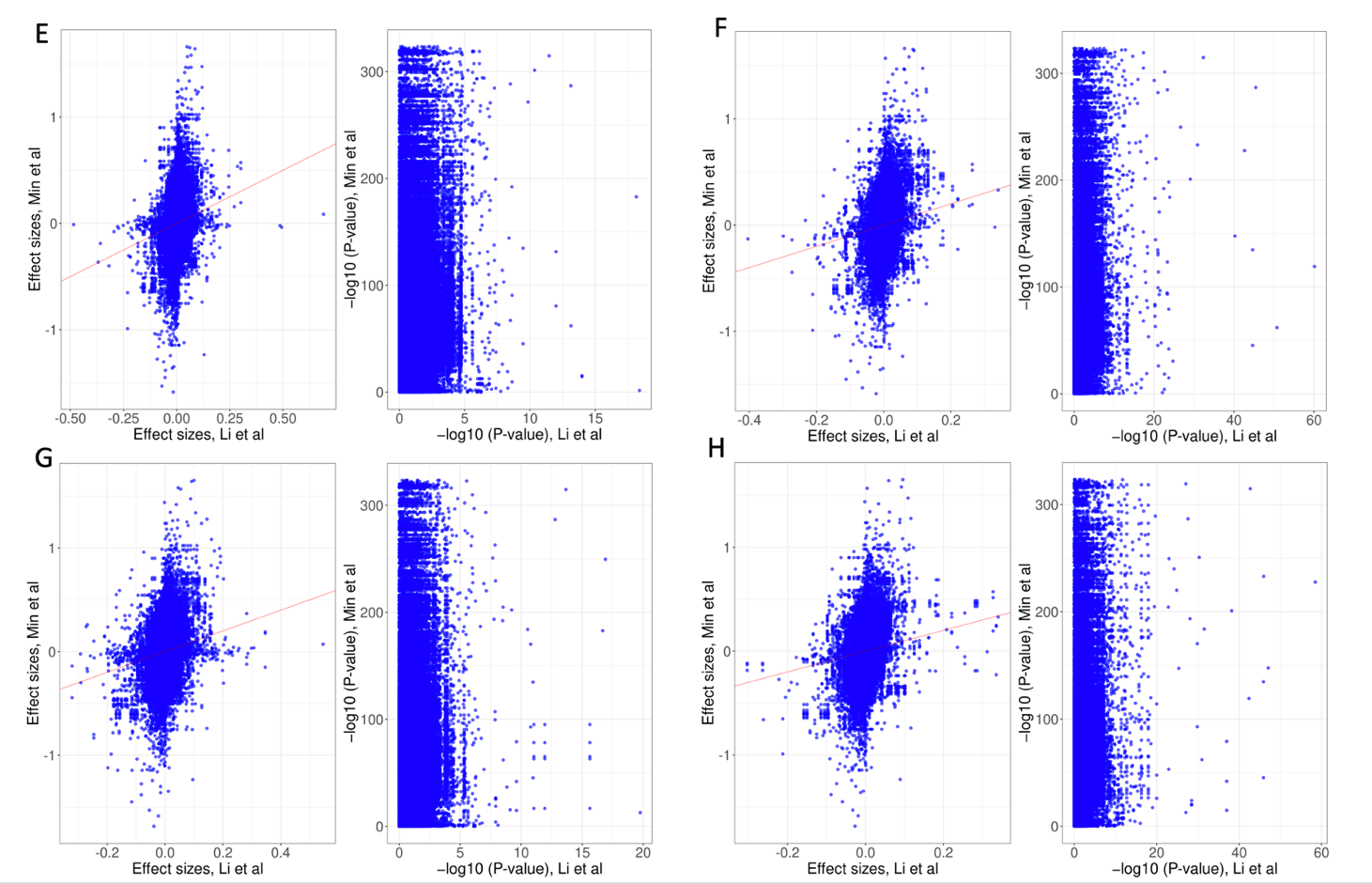


**Figure S6 Comparison of birthweight epigenome-wide association analysis results with or without controlling for common CpG mQTLs**

**S6A,** regression β coefficients a total of 114,897 shared CpGs between 450K and EPIC arrays were compared from two models in birthweight EWAS, one controlling for mQTL effects, the other one did not. The 1,928 CpGs that were significant were marked red, and CpGs that were not significant were marked turquoise. **6B,** negative log P values from these two models were compared, with significant CpGs marked red.


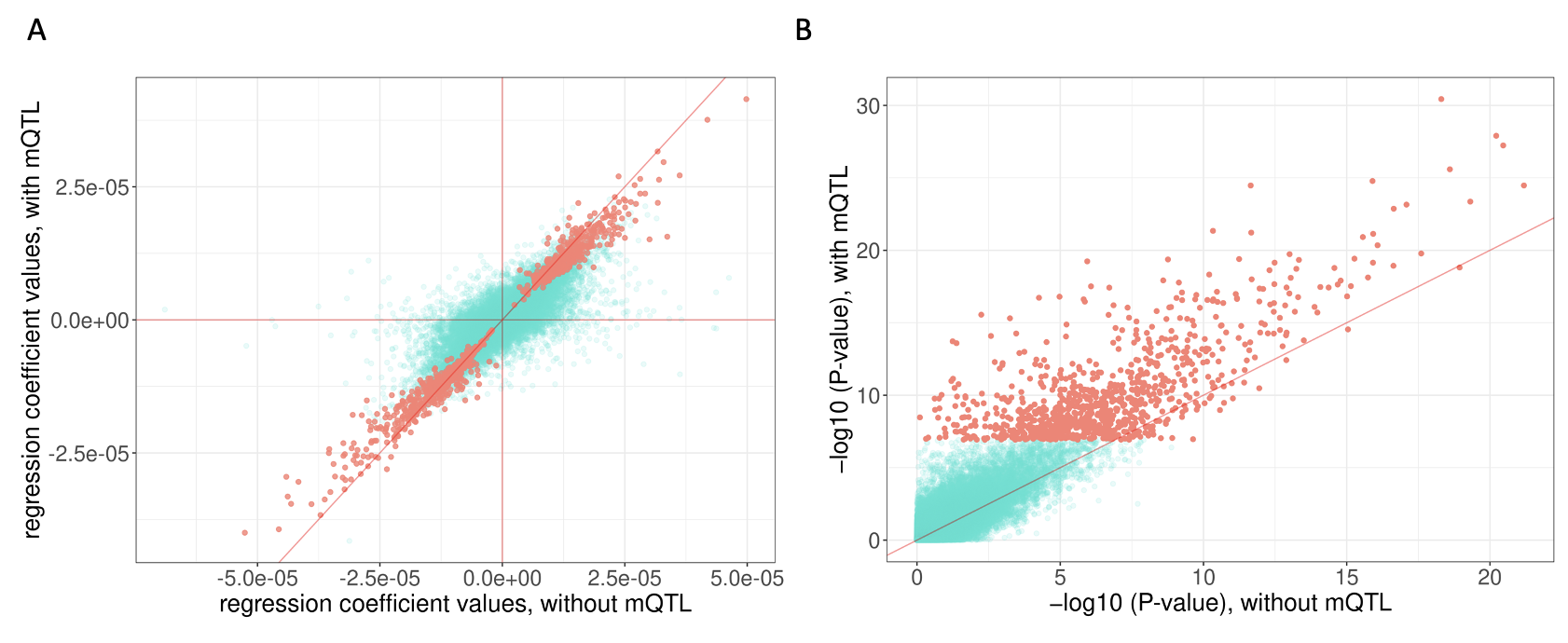


**Figure S7 Comparison of results from original and sensitivity EWAS models adding maternal weight gain as additional covariate**

Maternal weight gain during pregnancy was added as another covariate for those subjects with data available as a sensitivity analysis. Set 4 results were shown. **S7A**, Comparison of regression coefficient values. **S7B**, comparison of negative log P values.


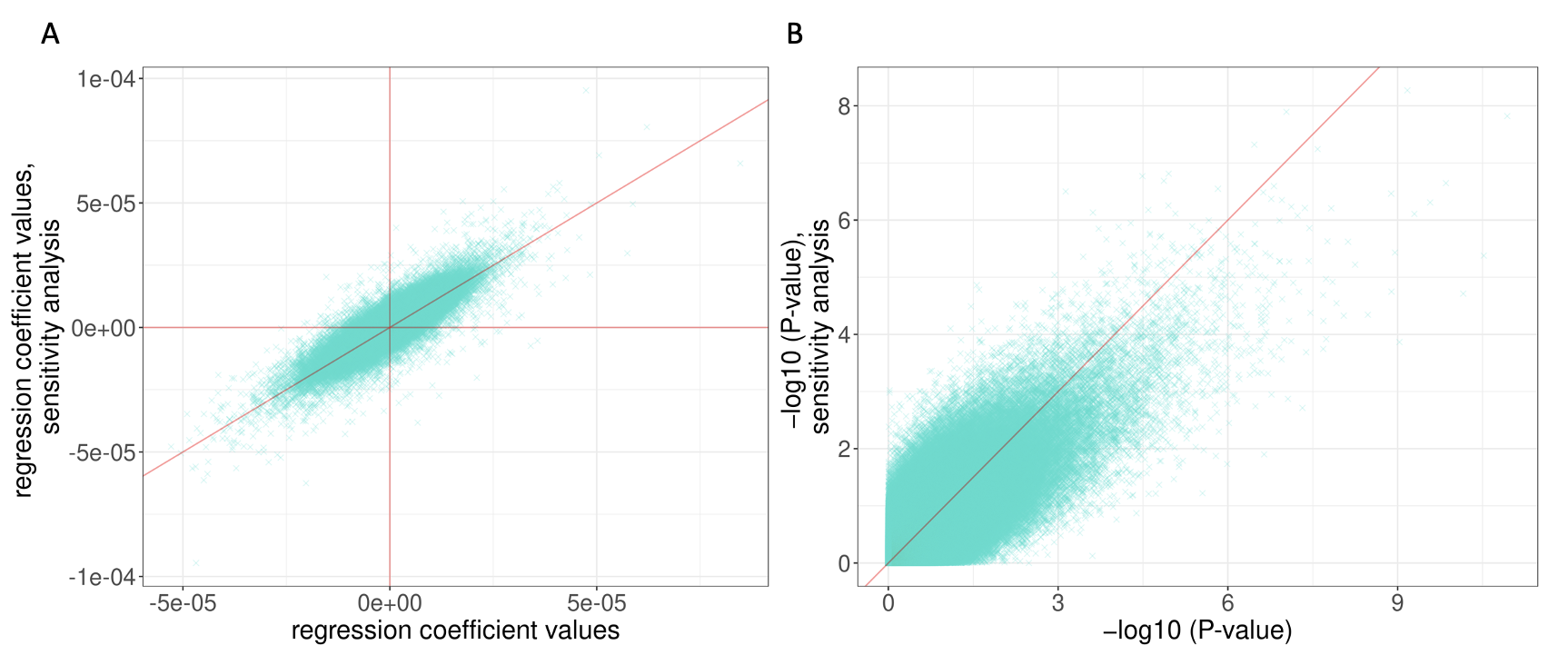

Supplement: Supplementary file 3 — Additional file 3. Supplement Figures S1–S7. [file 13148_2022_1385_MOESM3_ESM.docx]
